# Quantitative Measurements of Breast Density Using Magnetic Resonance Imaging: A Systematic Review and Meta-Analysis

**DOI:** 10.3390/jcm8050745

**Published:** 2019-05-24

**Authors:** Rooa Sindi, Cláudia Sá Dos Reis, Colleen Bennett, Gil Stevenson, Zhonghua Sun

**Affiliations:** 1 Discipline of Medical Radiation Sciences, School of Molecular and Life Sciences, Curtin University, Perth, WA 6845, Australia; rooa.sindi@postgrad.curtin.edu.au (R.S.); colleen.bennett@curtin.edu.au (C.B.); 2 School of Health Sciences (HESAV), University of Applied Sciences and Arts Western Switzerland (HES-SO), Av. de Beaumont 21, 1011 Lausanne, Switzerland; 3 CISP—Centro de Investigação em Saúde Pública, Escola Nacional de Saúde Pública, Universidade NOVA de Lisboa, 1600-560 Lisboa, Portugal; 4 Real Statistics, Bangor, County Down BT19 7PX, Northern Ireland, UK; real_statistics@btinternet.com

**Keywords:** magnetic resonance imaging, breast density, fibroglandular-tissue, systematic review and meta-analysis, cluster analysis, segmentation, FCM, breast-imaging protocol, non-contrast-enhanced T1-weighted

## Abstract

Breast density, a measure of dense fibroglandular tissue relative to non-dense fatty tissue, is confirmed as an independent risk factor of breast cancer. Although there has been an increasing interest in the quantitative assessment of breast density, no research has investigated the optimal technical approach of breast MRI in this aspect. Therefore, we performed a systematic review and meta-analysis to analyze the current studies on quantitative assessment of breast density using MRI and to determine the most appropriate technical/operational protocol. Databases (PubMed, EMBASE, ScienceDirect, and Web of Science) were searched systematically for eligible studies. Single arm meta-analysis was conducted to determine quantitative values of MRI in breast density assessments. Combined means with their 95% confidence interval (CI) were calculated using a fixed-effect model. In addition, subgroup meta-analyses were performed with stratification by breast density segmentation/measurement method. Furthermore, alternative groupings based on statistical similarities were identified via a cluster analysis employing study means and standard deviations in a Nearest Neighbor/Single Linkage. A total of 38 studies matched the inclusion criteria for this systematic review. Twenty-one of these studies were judged to be eligible for meta-analysis. The results indicated, generally, high levels of heterogeneity between study means within groups and high levels of heterogeneity between study variances within groups. The studies in two main clusters identified by the cluster analysis were also subjected to meta-analyses. The review confirmed high levels of heterogeneity within the breast density studies, considered to be due mainly to the applications of MR breast-imaging protocols and the use of breast density segmentation/measurement methods. Further research should be performed to determine the most appropriate protocol and method for quantifying breast density using MRI.

## 1. Introduction

Breast density, a measure of dense fibroglandular tissue relative to non-dense fatty tissue, is an independent risk factor for breast cancer [1,2,3]. Consistent with this risk relationship, women who have dense breasts have a likelihood of developing breast cancer that is fourfold higher than those with fatty breasts [4,5]. Most of the information regarding breast density has been acquired with two-dimensional imaging, which is mammography. However, the evaluation of breast density based on mammograms is limited due to the overlapping of tissues, variations in breast compression, and inappropriate positioning that lead to artefacts (skin folder) and inclusion of insufficient breast tissue [6,7]. These factors could affect mammography’s performance for precise, reliable measurements of small changes in breast density over brief timespans [8,9].

Magnetic resonance imaging (MRI), an alternative imaging modality in breast imaging can estimate the actual breast density value because it provides a three-dimensional volume assessment of breast tissue, with excellent contrast resolution in the differentiation between fibroglandular and fatty tissues [10,11,12,13]. Conventionally, breast density is assessed qualitatively using the American College of Radiology (ACR) Breast Imaging-Reporting and Data System (BI-RADS) atlas, which is a classification system commonly used for mammography, according to which density has four categories based on the amount of fibroglandular tissue: “(1) almost entirely fat, (2) scattered fibroglandular tissue, (3) heterogeneous fibroglandular dense and (4) extreme fibroglandular tissue” [14,15]. The interpretations of these four categories are also applied for MRI. Despite its long clinical success, the BI-RADS scoring atlas is subjective and varies between readers, even within the same reader [16]. To overcome a subjective assessment of breast density and to reduce inter- and intra-reader variability, different methods for quantitative breast density have been proposed, with a range of algorithms or methods reported in the literature [17,18,19,20,21,22]. Each of these methods were shown to have advantages and limitations through the use of semi-automatic thresholding and segmentation approaches for quantitative assessment of breast density.

There is no doubt that MRI is one of the most useful modalities for breast imaging and that the analysis of breast density in quantitative synthesis is a well-established approach. In spite of the fact that extensive research has been carried out on breast density measurements, no consensus has been reached about the optimal approach to quantify breast density using MRI. Therefore, the purpose of this review is to analyze the current methods for the quantitative assessment of breast density using MRI over the past decade of publications. Due to the expected heterogeneity of MRI scanning protocols, both systematic review and meta-analysis were performed to analyze the available studies.

## 2. Materials and Methods

This systematic review and meta-analysis were performed according to the Preferred Reporting Items for Systematic Reviews and Meta-Analysis (PRISMA) criteria [23,24]. No ethics committee approval was required.

### 2.1. Search Strategy and Eligibility Criteria

A systematic literature review was conducted of studies that analyzed breast density in a quantitative pattern using MRI. Briefly, a search for studies published between 1 January 2009 and 31 December 2018 was conducted in different databases: PubMed (MEDLINE, U.S. National Library of Medicine and National Institutes of Health, Bethesda, MD, USA), EMBASE (Elsevier, Amsterdam, The Netherlands), ScienceDirect (Elsevier, Amsterdam, The Netherlands), and Web of Science (Clarivate Analytics, Philadelphia, PA, USA) using the search terms detailed below.

Systematic search expressions were employed using MeSH (medical subject headings) in PubMed and the thesaurus in EMBASE, ScienceDirect, and Web of Science. A search structure was based on combining three main terms as follows: “breast density,” “quantitative analysis,” and “MRI.” The exact search expressions were “Breast Density” (MeSH term) OR “fibroglandular tissue” (Text word) OR “breast densit*” (Text word) OR “FGT” (Text word) OR “FT” (Text word) OR “fibroglandular densit*” (Text word) AND “Quantitative analysis” (Subject heading) AND “Magnetic Resonance Imaging” (MeSH term) OR “nuclear magnetic resonance imaging” (Text word) OR “MRI” (Text word) OR “magnetic resonance imaging” (Text word). The criteria for selecting the studies for eligibility were based on their title, abstract, and subsequently the full text, this was performed independently by two reviewers (R.S. and Z.S.). Studies addressing the quantitative analysis of breast density using MRI were considered eligible for inclusion and also studies on human subjects since 2009 had to be published in peer-reviewed journals and written in English. For study inclusion, the subjects must have undergone breast MRI studies and the breast density measurement method is known. Eligible studies were retrieved, and full manuscripts were read. No restricted conditions have been applied in terms of study characteristics, the purpose of study, and the results. Publications were only included in the analysis if the measurement of breast density had been performed in a quantitative manner regardless of the MRI technique or breast density segmentation/measurement method.

### 2.2. Data Extraction

On completing the eligibility screening, the process of data extraction from the included studies was carried out manually by the same two reviewers. Descriptive data were extracted for all variables as follows: the first author’s surname; year of publication; journal of publication; study type; total number of participants/patients; mean age; age range of participants/patients; MRI technique (pulse sequence/breast-imaging protocol and static magnetic field strength); and breast segmentation/measurement method. For each study analyzed, estimates of breast volume, fibroglandular-tissue volume and percentage breast density were recorded using descriptive statistics, arithmetic means and standard deviations, whenever appropriate. Due to the heterogeneous nature of this analysis, some of the included studies produced their results in a median and interquartile range (IQR). Accordingly, the researchers decided to stratify results and excluded them from the meta-analysis only.

### 2.3. Data Synthesis

The combinations of MRI techniques and the applied breast segmentation/measurement methods encountered in the studies were considered to be technologically heterogeneous. To address this issue and acquire more reasonable estimates, the analyses were stratified by breast segmentation method into three discrete groups (fuzzy c-mean clustering (FCM), FCM and nonparametric nonuniformity normalization (N3), and signal intensity thresholding). In each sub meta-analysis, the number of the included studies were selected on the basis of a degree of homogeneity of their breast density segmentation/measurement results.

### 2.4. Statistical Analysis

The measurement of breast density as ascertained by MRI using semi- or fully-automated segmentation method was assessed. The primary outcome was the percentage breast density (%BD). Data input for each study within a group consisted of the study size (N), the ‘raw’ study mean (i.e., with no re-scaling or standardization), and the study standard deviation. The data was analyzed by the “*metamean*” function in the “*meta*” package in the R system, Version 3.4.1 (http://www.r-project.org/). This facilitates the meta-analysis of a single arm trial, as opposed to the traditional two arm trial with a control group and a treatment group, equivalent to a one-way analysis of variance. A forest plot was generated, displaying the individual study (%BD) means with 95% confidence interval (CI) limits, inverse variance study weights, and the pooled mean and confidence limits. Heterogeneity of study means was assessed using Cochran’s Q-test, and heterogeneity of study variances was assessed with Bartlett’s test. A conclusion to pool studies requires both heterogeneity tests to be non-significant at the 5% level.

As an alternative to grouping the studies on a technological basis, a cluster analysis was run to investigate any similarities between studies with respect to two attributes, namely study mean and study standard deviation. The International Business Machines Statistical Package for the Social Sciences (IBM SPSS) Statistics software Version 25.0 was used for cluster analysis. The procedure provides for a wide selection of combinations of distance measures and clustering methods, but for the current application, the simplest of these was chosen, namely Euclidean distance and nearest neighbor agglomeration. This algorithm calculates a proximity matrix of distances between all possible pairs of studies and allocates the closest pair into a cluster, then examines the remaining clusters to identify which is the next nearest or whether there is a pair that are closer to one another, and so on.

## 3. Results

### 3.1. Literature Search

**Figure 1:**
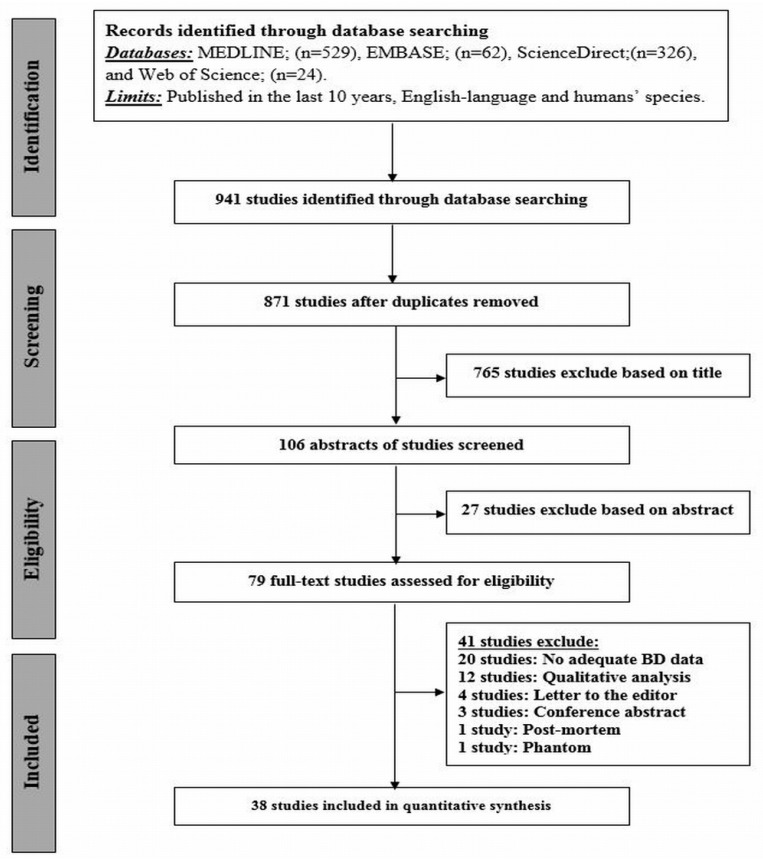
Preferred Reporting Items for Systematic Reviews and Meta-Analysis (PRISMA) flowchart of systematic review and meta-analysis of the excluded and included studies.

Figure 1 presents an overview of the systematic search of the literature through different databases. The complete search yielded 941 studies. After removing duplicates (n = 70), 871 were screened, based on their titles, which resulted in 765 being excluded, followed by 27 of the remaining studies being excluded on the basis of their abstracts. Of the remaining 79 studies, the full manuscript was retrieved and reviewed. Forty-one studies did not meet the selection inclusion criteria: no adequate breast density data (n = 20), qualitative analysis (n = 12), editorials (n = 4), conference abstracts (n = 3), post-mortem study (n = 1), and phantom study (n = 1). Finally, 38 studies attained the inclusion criteria [1,2,3,5,11,25,26,27,28,29,30,31,32,33,34,35,36,37,38,39,40,41,42,43,44,45,46,47,48,49,50,51,52,53,54,55,56,57] and were included in the analysis as shown in Table 1.

### 3.2. Systematic Review

**Figure 2:**
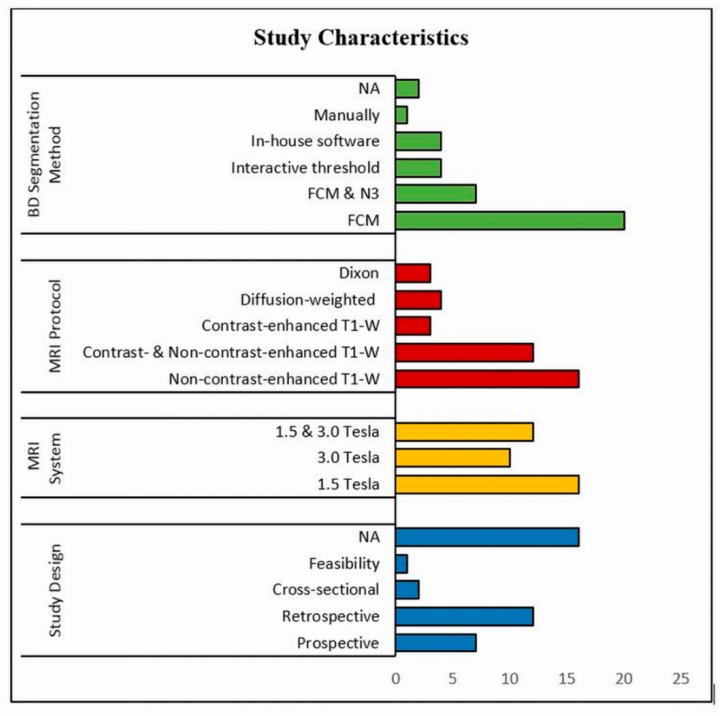
Flowchart of the study characteristics (study design, MRI system, MRI sequence, Breast Density (BD) segmentation method) of 38 studies.

Table 1 demonstrates some of the main characteristics of the 38 included studies, while Figure 2 shows details of the study design and MRI system used in these studies. Several MRI sequences were used to enable the precise differentiation between adipose and fibroglandular tissues; of these, non-contrast-enhanced T1-weighted was widely used either with 2D spin echo or 3D gradient echo. In fact, 16 studies (41.03%) used non-contrast-enhanced T1-weighted [1,2,26,27,28,29,31,33,35,44,45,48,49,50,51,53], while in 12 studies (30.77%) non-contrast-enhanced images were integrated with contrast-enhanced images [25,36,37,38,39,40,41,42,43,44,47,49]. In terms of breast density segmentation/measurement, the majority of the studies (20 studies; 51.28%) used FCM clustering algorithm [1,2,11,25,26,27,28,29,31,32,33,34,35,36,37,38,39,40,41,42], while 7 studies (17.95%) used FCM and N3 algorithm [45,46,47,48,49,50,51], 4 studies (10.26%) interactive thresholding algorithm [3,5,52,53], 4 studies (10.26%) in-house customized software [29,53,54,55], one study (2.56%) manual software [57]; however, two studies did not provide the information [43,44].

Among the thirty-eight studies included in the systematic review and meta-analysis, 21 studies qualified for meta-analysis since they reported the percent breast density using an identical expression of measurement and dispersion [1,3,11,25,26,27,28,29,30,31,32,36,37,38,45,48,49,50,53,54,55] (Table 2). However, for the remaining 17 studies, the percent breast density was reported in different format: in eight of these studies, it was defined as a median and interquartile range (IQR) [2,39,40,41,42,43,44,47], and in the other nine, it was reported either in different measurement unit or the subject’s sets were not independent, due to multiple usage [5,33,34,35,46,51,52,56,57]. To perform the meta-analysis precisely, all the measured quantities should be reported in an identical expression of measurement and dispersion, thus we decided to exclude them from the meta-analysis.

### 3.3. Subgroup Analyses

**Figure 3:**
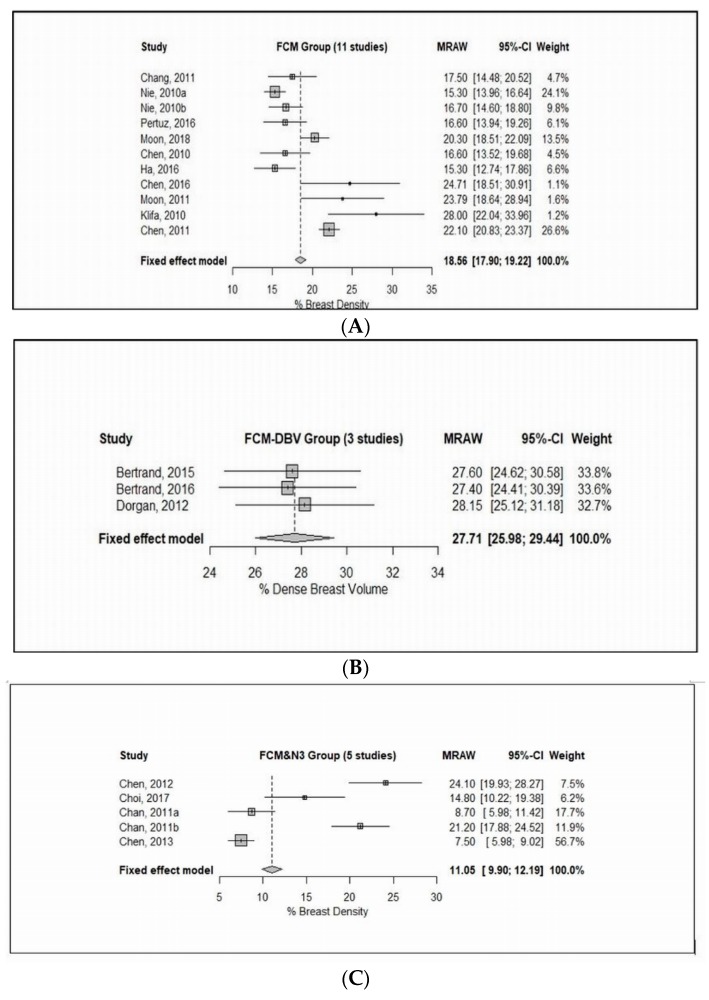

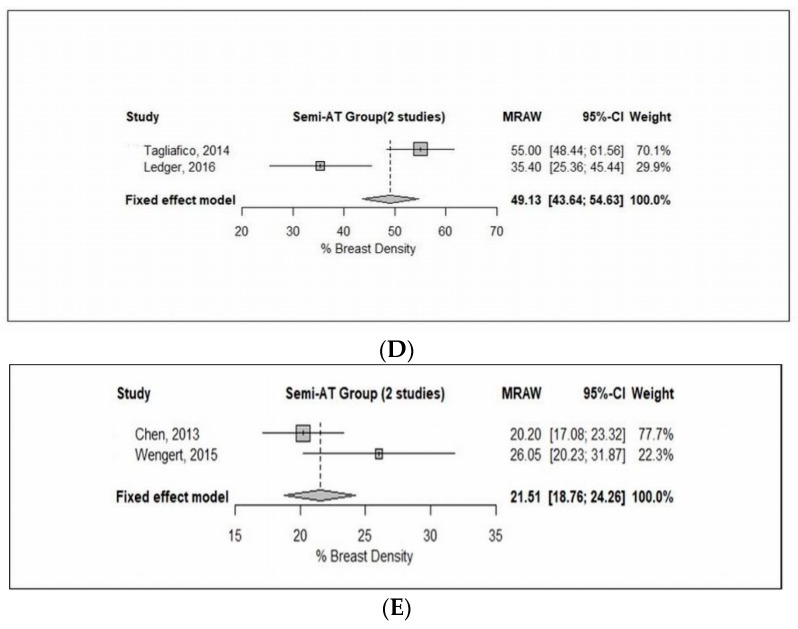
Forest plot of the study means, and 95% confidence limits of the breast density among 21 included studies in the subgroup meta-analyses. (**A**) Fixed effect meta-analysis of the fuzzy c-mean clustering (FCM) group of studies of % breast density. (**B**) Fixed effect meta-analysis of the FCM group of studies of % dense breast volume. (**C**) Fixed effect meta-analysis of the FCM and N3 group of studies of % breast density. (**D**) Fixed effect meta-analysis of the semi-automated threshold group of studies of % breast density. (**E**) Fixed effect meta-analysis of the semi-automated threshold group of studies of % breast density.

The final inclusion consisted of a total of twenty-one studies in the meta-analyses; the forest plots and pooled results are shown in Figure 3.

#### 3.3.1. Fuzzy C-mean Clustering (FCM)

The FCM subgroup consisted of 13 studies, of which, 10 studies reported breast density as a percentage breast density (% BD) [1,11,25,26,27,28,29,30,31,32], whereas 3 studies as a percentage of the dense breast volume (% DBV) [36,37,38]. On one hand, 10 studies with inclusion of 634 patients were included, as Figure 3A shows, there is a wide range of mean values as well as standard deviation (SDs) from those studies, which indicated enormous heterogeneity among study means (Cochran’s Q test: *X*^2^ = 86.93, *P* < 0.0001). Indeed, there is a substantial heterogeneity among study variances (Bartlett’s test: *X*^2^ = 110.59, *P* < 0.0001). On the other hand, three studies with inclusion of 528 patients were analyzed. Figure 3B shows there is a high level of homogeneity among study means (Cochran’s Q test: *X*^2^ = 0.13, *P* = 0.94), and a high level of homogeneity among study variances (Bartlett’s test: *X*^2^ = 0.12, *P* = 0.94), which would be expected as those studies used an identical combination of MR technique and breast density segmentation/measurement approach.

#### 3.3.2. FCM and Nonparametric Nonuniformity Normalization (N3)

The FCM and N3 subgroup included 4 studies with inclusion of 126 patients [45,48,49,50], as Figure 3C shows, there is a wide range of mean values as well as SDs from those studies, which indicated tremendous heterogeneity among study means (Cochran’s Q test: *X*^2^ = 99.94, *P* < 0.0001). Indeed, there is a substantial heterogeneity among study variances (Bartlett’s test: *X*^2^ = 45.41, *P* < 0.0001), which would be expected as those studies used different MR breast-imaging protocols.

#### 3.3.3. Interactive Semi-Automated Threshold

Two studies [3,54] comprising of 58 patients were included in the analysis, which indicated a considerable heterogeneity among study means (Cochran’s Q test: *X*^2^ = 10.26, *P* = 0.0014). In contrast, there was no evidence of heterogeneity among study variances (Bartlett’s test: *X*^2^ = 1.61, *P* = 0.2072).

On the other hand, two studies with inclusion of 67 patients [53,55] were analyzed as shown in Figure 3E, there was no evidence of heterogeneity among study means (Cochran’s Q test: *X*^2^ = 3.01, *P* = 0.0825), which would be expected as those studies used the same MRI technique and breast density measurement. However, there is a substantial heterogeneity among study variances (Bartlett’s test: *X*^2^ = 18.84, *P* < 0.0001).

### 3.4. Cluster Analysis

**Figure 4:**
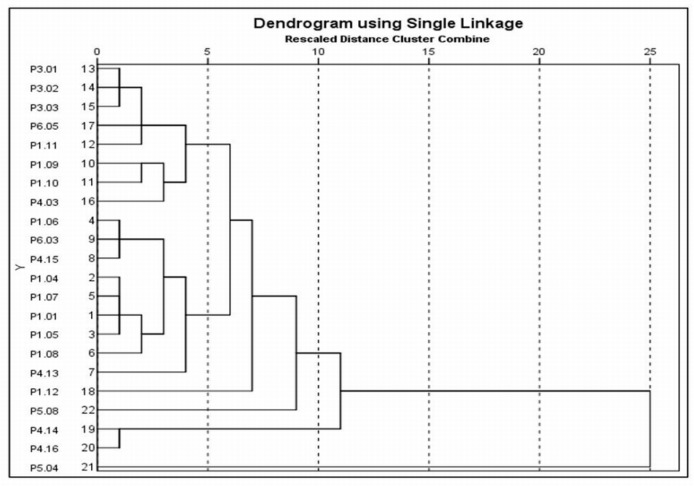
Dendrogram clustering analysis using “Single Linkage” method of the study means, and study SDs among 21 included studies in the subgroup meta-analyses.

**Figure 5:**
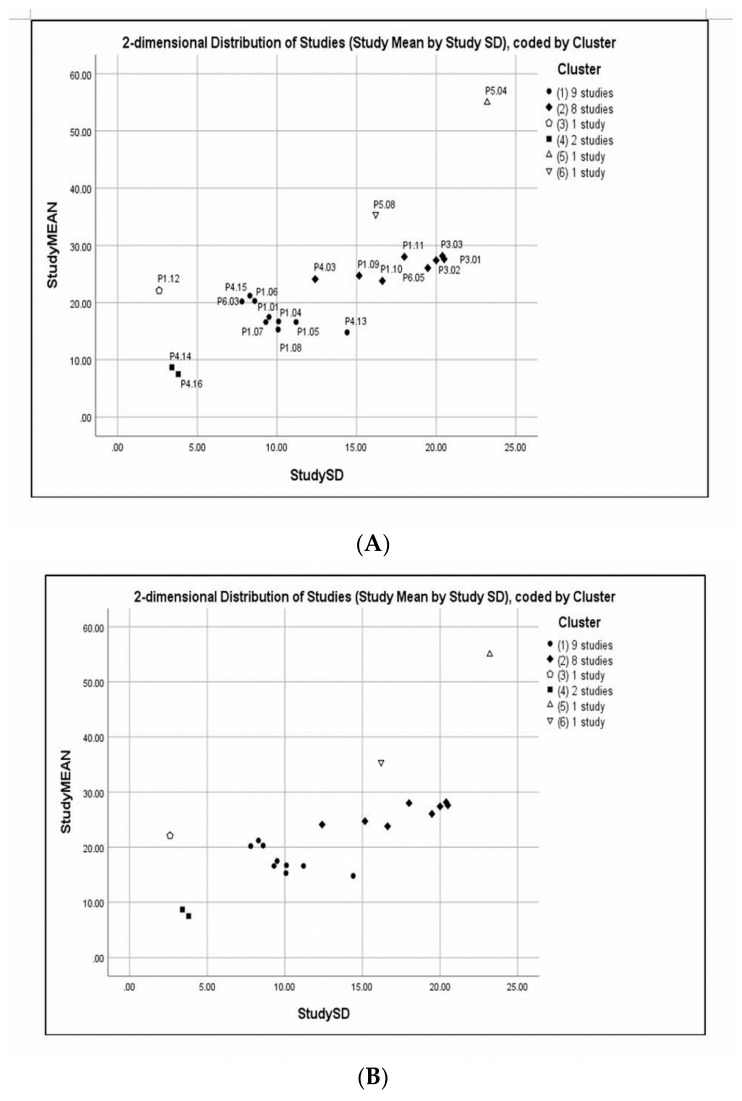
Scatter plot of the study means versus SDs using 6-clusters memberships of the 21 included studies in the subgroup meta-analyses. Legend indicates the number of studies in each cluster, solid fill represents clusters with two or more studies, while open markers represent singleton study. Scatterplot of study means versus SDs with study codes (**A**) and without study codes (**B**).

The results obtained from the clustering analysis “Dendrogram using Single Linkage” are shown in Figure 4. From this data, it can be seen that a hierarchical diagram showing various distances (0–25) at which studies joined various groups. On that basis, six clusters were identified. A list of cluster membership, study means, SDs, and coefficient of variations (CVs) (expressed as a percentage) is shown in Table 3. A scatter plot of the study means versus SDs is shown in Figure 5, the legend in the scatter plot indicates the number of studies in each cluster. Cluster markers with solid fill indicate clusters with two or more studies, whereas open markers indicate singletons. Cluster 1 included nine studies that analyzed breast density with a combination of contrast and non-contrast T1-weighted either with 2D spin echo or 3D gradient echo; however, Choi [49] used diffusion-weighted scanning technique. From the data in Table 3 (Cluster 1), it is apparent that the CVs are varied in value, but in Choi’s study [49] the CV is almost 100% because of the mean and SD are almost identical. In contrast, the CVs for Chan [48] and Chen [53] are much lower than the rest of the included studies, largely because of the small SDs and the breast segmentation methods being used which are FCM and N3 and interactive semi-automated threshold algorithms, respectively.

In contrast, cluster 2 consisted of 8 studies that assessed breast density with a combination of contrast- and non-contrast-enhanced T1-weighted with 3D gradient echo, however, Chen [31] and Chen [45] used non-contrast- and contrast-enhanced T1-weighted with 2D spin-echo, respectively. Indeed, Chen [45] analyzed breast density using FCM and N3 algorithms. From the data in Figure 5 and Table 3 (Cluster 2), it is apparent that the CVs are almost within a closed range except for Chen [45] where the CV is much lower than the remaining studies because of the small SD and the breast segmentation method that previously mentioned. Also, Wengert [55] used Dixon method as a technical protocol for breast-imaging, although they measured the breast density using in-house customized software, the mean and SD are not different to the other included studies. The most striking result to emerge from the data in Figure 5 and Table 3 (Cluster 3–6) is the Chen [1] study (i.e., Cluster 3), although it used non-contrast-enhanced T1-weighted with 3D gradient echo and analyzed breast density by FCM algorithm, the CV (11.67%) is much lower than the remaining studies, mainly because of the small SD.

Cluster 4 included two studies Chan [48] and Chen [50] that assessed breast density using FCM and N3 algorithms and non-contrast-enhanced T1-weighted with 3D gradient echo. As can be seen from the data in Figure 5 and Table 3 (Cluster 3–6) the study means and SDs are not different. In contrast to this Cluster 5, the Tagliafico study [3] used 3D contrast-enhanced T1-weighted gradient echo sequence and analyzed the breast density by semi-automated interactive threshold, in particular, (MedDensity). As Figure 5 and Table 3 (Cluster 3–6) show, the study mean is much higher than the remaining studies, largely because of the technical method used. Finally, cluster 6 consisted of Lodger [54], this is the only study that used proton density weighted sequence. Detailed information of clustering membership, study means, SDs, and CVs is shown in Table 3, Figure 4 and Figure 5.

**Figure 6:**
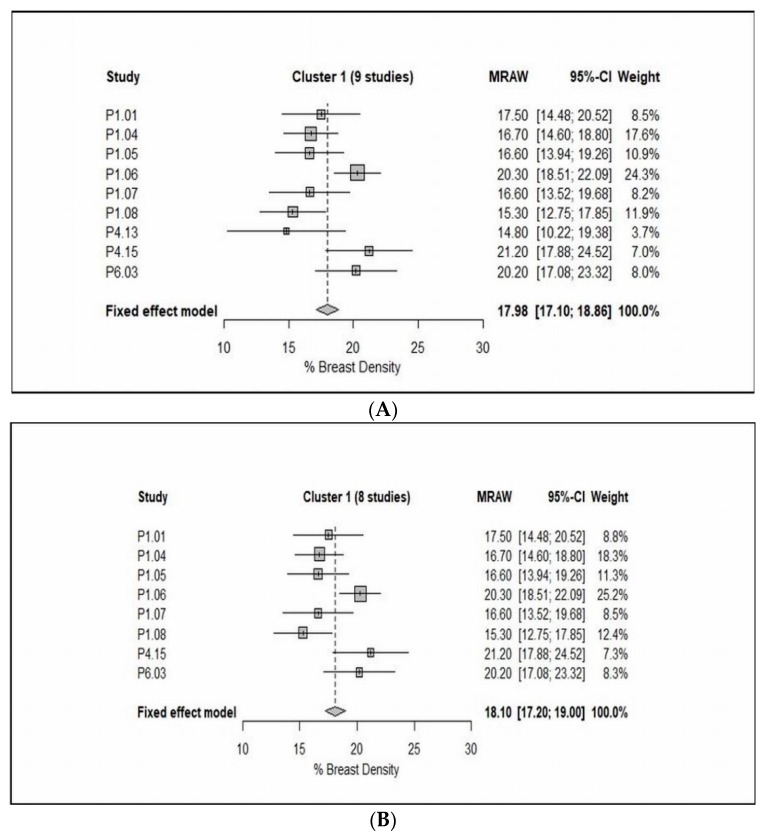
Forest plot of the study means, and 95% confidence limits of the studies in Cluster 1 with/without P4.13 (Choi, 2017) of % breast density. (**A**) Fixed effect meta-analysis of the studies in Cluster 1 (9 studies) of % breast density. (**B**) Fixed effect meta-analysis of the studies in Cluster 1 (8 studies) of % breast density.

**Figure 7:**
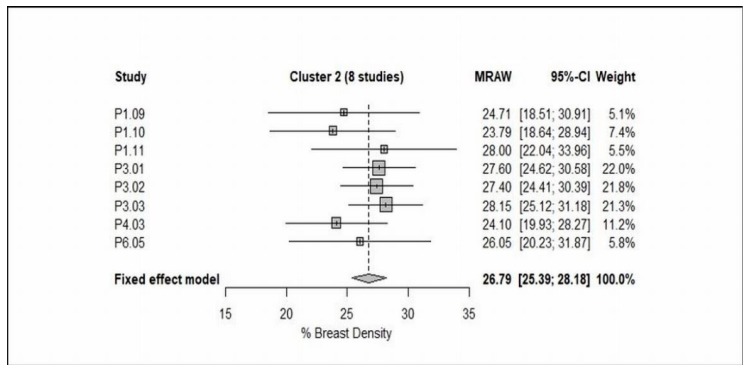
Forest plot of the study means, and 95% confidence limits of the studies in Cluster 2 (8 studies) of % breast density.

Switching from technology groupings of studies to groupings identified by the cluster analysis, meta-analysis of cluster 1 revealed that the study means, and study variances are both heterogeneous (Cochran’s test for heterogeneity of study means, *X*^2^ = 22.26, *P* = 0.0045, and Bartlett’s test for heterogeneity of study variances, *X*^2^ = 21.47, *P* = 0.0060, see Figure 6A). When Choi [49] was excluded (because of the very large CV), the cluster has improved somewhat, which would be expected as this study used different protocols (i.e., diffusion-weighted imaging). It can be seen from the data in Figure 6B that the study variances are no longer heterogeneous (*X*^2^ = 8.84, *P* = 0.2641), although the study means remain heterogeneous (*X*^2^ = 19.54, *P* = 0.0066). In contrast, meta-analysis of cluster 2 indicated that the study means are not heterogeneous (*X*^2^ = 4.77, *P* = 0.6874), while the study variances are mildly heterogeneous (*X*^2^ = 15.54, *P* = 0.0206, see Figure 7).

## 4. Discussion

The present systematic review and meta-analysis was performed to analyze the current studies on quantitative breast density using MRI and to determine the most appropriate technical/operational protocol. Through reviewing 38 studies from the literature, despite many methods and protocols available, no gold standard has been established with a wide range of heterogeneous methods or protocols used in these studies. To the best of our knowledge, this is the first comprehensive systematic review and meta-analysis of pooling the results of all breast density segmentation/measurement methods using MRI data. The analysis indicated that the non-contrast-enhanced T1-weighted acquisition was commonly utilized among all MR breast-imaging protocols. Another important finding of this analysis was that the FCM is the most frequently used algorithm amongst the breast density segmentation/measurement methods. Also, the results showed that a high level of heterogeneity was mainly associated with the breast-imaging protocols and the breast density segmentation/measurement methods.

Further attempts have been made by using clustering methods and meta-analysis to identify groups of studies which are as homogeneous as possible within groups and as heterogeneous as possible between groups. The included studies were grouped together into clusters based on their nearest neighbor Euclidean distances. On that basis, clusters 1 and 2 were considered as the most valuable results. Briefly, cluster 1 consisted of 9 studies [25,26,27,28,29,30,48,49,53], as shown from the data in Table 3 and Figure 6A that the CVs are varied in value, but in Choi [49] the CV is almost 100% because of the mean and SD are almost identical. This result may be explained by the fact that among the 8 studies [11,31,32,36,37,38,45,55], the breast-imaging protocol was a combination of contrast- and non-contrast-enhanced T1-weighted either with 2D spin echo or 3D gradient echo, while in Choi [49] the MRI protocol used was diffusion-weighted imaging. Consequently, it is advisable to exclude it from the meta-analysis to reduce the heterogeneity within cluster 1. Consistent with this hypothesis, the results have improved in somewhat, even though the study variances are not heterogeneous (*P* > 0.05), the study means are heterogeneous (*P* < 0.05) (Figure 6B). Although exclusion of Choi [49] did not reduce the heterogeneity, these results should be interpreted with caution. The discrepancy could be largely attributed to that although the MR breast-imaging protocols are not dissimilar (i.e., contrast- and non-contrast-enhanced T1-weighted), the breast segmentation/measurement methods are vice versa (i.e., FCM, FCM and N3, and in-house customized software). In contrast, cluster 2 included 8 studies, in 3 of these studies the breast density was reported as a (%DBV), while the remaining as a (% BD). Among these studies, the contrast- and non-contrast-enhanced T1-weighted was often used. From the data in Table 3 and Figure 7, it is apparent that the study means are not dissimilar (*P* > 0.05), although the study variances are heterogeneous (*P* < 0.05). Among the 21 studies included in the cluster analysis, although the fixed effect meta-analysis of cluster 2 has improved slightly, the heterogeneity within group still exist. There are two likely causes for this heterogeneity: the applied MR breast-imaging protocol and the used breast density segmentation/measurement methods.

Although the study has successfully confirmed the variation in the breast density segmentation/measurement methods using MRI data, the findings are subject to several limitations. First, the heterogeneity of study aims, the study design utilized, and the technical/operational methods applied, for instance, the MR breast-imaging protocol, MR scanner manufacturer, and the static magnetic field strength present challenges for performing the meta-analysis. Second, the breast density segmentation/measurement algorithm used is another limitation. Although we classified the included studies into discrete subgroups (i.e., FCM, FCM and N3, and interactive semi-automated threshold), and applied stratified analyses, the heterogeneity remains. Third, the definition of the breast density was inconsistent because some studies reported it as a percentage of dense breast volume, while the others as a percentage of breast density. Fourth, among the 38 studies included in this analysis, only 21 studies were eligible for meta-analysis due to the statistical requirements for the input values that should be in identical expression of measurement and dispersion. In addition, some of the included studies used the same set of the subject multiple times for different purpose and feature. Even though we decided to rectify the issue by selecting one of the results of data at random, or by any meaningful clinical criterion, the heterogeneity continues to exist. Notwithstanding these limitations, the study further supports the idea of developing a standard MRI protocol for the quantitative assessment of breast density.

Future research can be suggested according to findings of this review. A recent study has reported the feasibility of creating a realistic 3D printed breast phantom for quality control purpose [58]. Thus, we consider 3D printing technique can be used to develop a patient-specific 3D printed breast phantom with different amounts of breast composition to quantify the volume of FGT. Further, the 3D printed model can be used to examine several MR breast-imaging protocols not only to measure the breast density but also to assess the impact of implementing various image quality parameters (i.e., FOV, matrix size and slice thickness) on the segmentation/measurement of breast density. Finally, the accuracy of different breast density/FGT segmentation methods can be determined.

## 5. Conclusions

This systematic review and meta-analysis confirms and substantiates the variation among the breast density segmentation/measurement methods using MRI. Furthermore, subgroup meta-analyses and further clustering methods indicated that a significant heterogeneity within and between groups exist. The analysis confirmed that the non-contrast-enhanced T1-weighted acquisition was commonly utilized among all MR breast-imaging protocols and the FCM is the most frequently used algorithm amongst the breast density segmentation/measurement methods. Future work will need to determine the most appropriate protocol and method for quantifying breast density using MRI.

## Figures and Tables

**Table 1:** Characteristics of the included studies in the systematic review and meta-analysis.

Author, Year of Publication	Study Design	Study Participants	Age Range, Average (Years) or Mean ± SD	MR Scanner Manufacturer, Field Strength (Tesla)	MRI Sequence	Orientation, Slice No.	TR/TE (ms)	FOV (cm)	Slice Thickness (mm)	Matrix Size	Flip Angle (_°_)	Breast Coil	Segmentation Method
Chang, 2011 [25]	Retro.	38	28–82, 48	Philips, 3.0	Fat-suppressed 3D SPAIR	Axial, 160	6.20/1.26	3.01–38.0	1.0	480 × 480	12	NA	FCM
					Non-fat-suppressed 2D TSE	Axial, 84	800/8.6	31.0–38.0	2.0	480 × 480	90	NA	
Nie, 2010 [26]	NA	230	50 ± 11.0	Philips, 1.5	Non-fat-suppressed 3D SGRE (T1W)	Axial, 32	8.1/4.0	31.0–38.0	4.0	256 × 256	20	NA	FCM
Pertuz, 2016 [27]	Retro.	68	24–82, 52	Siemens, 1.5	Non-fat-suppressed (T1W)	NA	NA	NA	2.4–3.5	512 × 512	NA	NA	FCM
Moon, 2018 [28]	Retro.	98	51.81 ± 11.08	GE, 1.5	Non-fat-suppressed (T1W)	Axial	6.2/2.1	20.0	1.0	512 × 217	NA	NA	FCM
Chen, 2010 [29]	Retro.	35	45 ± 7	Philips, 1.5	Non-fat-suppressed 3D SGRE (T1W)	Axial, 32	8.1/4.0	38.0	3.0–4.0	256 × 128	20	Dedicated 4-channel phased array	FCM
Chen, 2016 [31]	Retro.	23	40.5 ± 8.2	Philips, 3.0	Non-fat-suppressed 2D TSE (T1W)	Axial, 90	654/9.0	33.0	2.0	328 × 384	NA	NA	FCM
Moon, 2011 [32]	Retro.	40	50.9 ± 9.4	GE, 1.5	Fat-suppressed 3D GRE (T1W) (VIBRANT)	Sagittal, 144–192	6.1/2.5	19.0	1.5	512 × 512	NA	NA	FCM
Klifa, 2010 [11]	Retro.	35	28–59, 43	GE, 1.5	Fat-suppressed 3D Fast GRE (T1W)	Axial, 60	8.0/4.2	NA	2.0	NA	20	Dedicated bilateral phased array	FCM
Chen, 2011 [1]	Retro.	16	33–51, 43	GE, 1.5	Non-fat-suppressed 3D (T1W)	Axial, 56	7.4/3.3	30	2.0	512 × 512	NA	Dedicated 8-channel bilateral	FCM
Nie, 2010 [33]	Retro.	50	NA	Philips, 1.5	Non-fat-suppressed 3D GRE (T1W)	Axial, 32	8.1/4.0	38.0	4.0	256 × 128	20	NA	FCM
Kim, 2014 [34]	Retro.	80	27–68, 44	GE, 1.5	Fat-suppressed 2D FSE (T2W)	Sagittal	5500-7150/82	20.0	1.5	256 × 160	NA	Dedicated 8-channel bilateral	FCM
					Fat-suppressed 3D Fast SGRE (T2W)	Sagittal	6.2/2.5	20.0	1.5	256 × 160	10	Dedicated 8-channel bilateral	
Nie, 2010 [35]	Retro.	321	54 ± 12	Philips, 1.5	Non-fat-suppressed 3D SGRE (T1W)	Axial, 32	8.1/4.0	32.0–38.0	4.0	256 × 128	20	Dedicated 4-channel phased-array	FCM
Wang, 2013 [2]	Retro.	99	47.2 ± 12.1	GE, 1.5/3.0	Non-fat-suppressed (T1W)	Axial	NA	NA	2.0	NA	NA	Dedicated bilateral phased-array	FCM
Bertrand, 2015 [36]	Pros.	182	25–29	NA, 1.5/3.0	Non-fat- and fat-suppressed 3D Fast GRE (T1W)	Axial and Coronal	NA	32.0–40.0	NA	NA	NA	Dedicated RF coil	FCM
Bertrand, 2016 [37]	Pros.	172	25–29	NA, 1.5/3.0	Non-fat- and fat-suppressed 3D Fast GRE (T1W)	NA	NA	32.0–40.0	NA	NA	NA	Dedicated RF coil	FCM
Dorgan, 2012 [38]	Retro.	174	25–29	NA, 1.5/3.0	Non-fat- and fat-suppressed 3D Fast GRE (T1W)	Axial and Coronal	NA	32.0–40.0	NA	NA	NA	Dedicated RF coil	FCM
Gabriel, 2013 [39]	NA	182	25–29	NA, 1.5/3.0	Non-fat- and fat-suppressed 3D Fast GRE (T1W)	Axial and Coronal	NA	32.0–40.0	NA	NA	NA	Dedicated RF coil	FCM
Jung, 2015 [40]	Pros.	180	25–29	NA, 1.5/3.0	Non-fat- and fat-suppressed 3D Fast GRE (T1W)	Axial and Coronal	NA	32.0–40.0	NA	NA	NA	Dedicated RF coil	FCM
Jung, 2016 [41]	Pros.	177	25–29	NA, 1.5/3.0	Non-fat- and fat-suppressed 3D Fast GRE (T1W)	Axial and Coronal	NA	32.0–40.0	NA	NA	NA	Dedicated RF coil	FCM
Dorgan, 2013 [42]	C.S.	176	27.0–27.3, 27.2	NA, 1.5/3.0	Non-fat- and fat-suppressed 3D Fast GRE (T1W)	Axial and Coronal	NA	32.0–40.0	NA	NA	NA	Dedicated RF coil	FCM
Jung, 2015 [43]	Pros.	177	25–29	NA, 1.5/3.0	Non-fat- and fat-suppressed 3D Fast GRE (T1W)	Axial and Coronal	NA	NA	NA	NA	NA	Dedicated RF coil	NA
Jones, 2015 [44]	C.S.	172	25–29	NA, 1.5/3.0	Non-fat- and fat-suppressed 3D Fast GRE (T1W)	Axial and Coronal	NA	NA	NA	NA	NA	Dedicated RF coil	NA
Chen, 2012 [45]	Pros.	34	20–64, 35	GE, 1.5	Non-fat-suppressed 2D FSE (T1W)	Axial	607/9.0	38.0	2.0	256 × 192	NA	Dedicated 8-channel bilateral	FCM and N3
				GE, 3.0	Non-fat-suppressed 2D FSE (T1W)	Axial	650/9.0	38.0	2.0	256 × 192	NA	Dedicated 8-channel bilateral	
				Philips, 3.0	Non-fat-suppressed 2D FSE (T1W)	Axial	650/9.0	33.0	2.0	328 × 384	NA	Dedicated 16-channel bilateral	
				Siemens, 1.5	Non-fat-suppressed 2D FSE (T1W)	Axial	650/9.8	33.0	2.0	330 × 384	20	Dedicated 4-channel bilateral	
Chen, 2015 [46]	NA	32	22–53, 41	Siemens, 1.5	Non-fat-suppressed 2D FSE (T1W)	Axial	650/9.8	33.0	2.0	256 × 256 and 512 × 512	NA	Dedicated 4-channel bilateral	FCM and N3
Chen, 2013 [47]	NA	44	28–82, 47	Philips, 3.0	Non-fat-suppressed 2D TSE (T1W)	Axial	800/8.6	31.0–38.0	2.0	480 × 480	90	Dedicated 4-channel bilateral	FCM and N3
					Fat-suppressed 3D GRE (T1W)	Axial	6.2/1.26	31.0–36.0	2.0	480 × 480	12	Dedicated 4-channel bilateral	
Chan, 2011 [48]	NA	30	Pre: (N = 24)	Siemens, 1.5	Non-fat-suppressed 3D GRE (T1W)	Axial	11/4.7	35.0	2.0	256 × 256	20	4-channel dual-mode	FCM and N3
			23–48, 29										
			Post: (N = 6)										
			51–61, 57										
Choi, 2017 [49]	Retro.	38	32–79, 45	Philips, 3.0	Non-fat-suppressed SE (T1W)	Axial	620/10	20.0–34.0	3.0	332 × 332	NA	Dedicated 7-channel bilateral	FCM and N3
					STIR and SE-EPI (DW)	Axial	3265/54	35.0	4.0	288 × 288	90	Dedicated 7-channel bilateral	
Chen, 2013 [50]	NA	24	23–48, 29	Siemens, 1.5	Non-fat-suppressed 3D Fast GRE (T1W)	Axial	11/4.7	35.0	2.0	256 × 256	20	4-channel dual-mode	FCM and N3
Clendenen, 2013 [51]	NA	9	24–31	Siemens, 3.0	Non-fat-suppressed 3D VIBE (T1W)	Axial	4.19/1.62	26.9 × 20.2 × 28.8	0.6 × 0.6 × 1	448 × 336 × 288	12	Dedicated 7-channel bilateral	FCM and N3
					3-Point Dixon Non-fat-suppressed 3D FLASH (T1W)	Axial	7.6/3.37, 4.17. 4.96	NA	0.88 × 0.88 × 1.5	NA	10	Dedicated 7-channel bilateral	
McDonald, 2014 [52]	Retro.	103	47 ± 11	Philips, 3.0	EPI-Parallel Imaging (DWI)	NA	5336/61	36.0	5.0	240 × 240	NA	Dedicated 16 channel bilateral	Semi-automated Interactive Threshold
Tagliafico, 2013 [5]	Pros.	48	35–67, 41	GE, 3.0	3D Fast SGRE and Fat-suppressed 3D GRE (T1W) (VIBRANT)	NA	6.2/3.0	NA	NA	350 × 350	10	Dedicated 8-channel bilateral	Semi-automated Interactive Threshold
					IDEAL	NA	4380/130.872	NA	NA	360 × 360	90	Dedicated 8-channel bilateral	
Tagliafico, 2014 [3]	NA	48	35–67, 41	GE, 3.0	TSE (T1W)	NA	600/9.0	NA	4.0	350 × 350	90	Dedicated 8-channel bilateral	Semi-automated Interactive Threshold
					TSE (T2W)	NA	5200/103	NA	4.0	350 × 350	90	Dedicated 8-channel bilateral	
					Fat-suppressed 3D GRE (T1W) (VIBRANT)	NA	6.2/3.0	NA	1.2	350 × 350	10	Dedicated 8-channel bilateral	
					IDEAL	NA	4380/130	NA	1.2	360 × 360	90	Dedicated 8-channel bilateral	
Chen, 2013 [53]	NA	24	23–48, 29.4	Siemens, 1.5	Non-fat-suppressed 3D GRE (T1W)	Axial	11/4.7	35.0	2.0	256 × 256	20	NA	Semi-automated Interactive Threshold
Ha, 2016 [30]	Retro.	60	54.2	GE, 1.5/3.0	Fat-suppressed Fast SGRE (T1W)	Axial	17/2.4	18.0-22.0	2.0	256 × 192	35	8-channel breast array	Semi-automated (In-house software)
Ledger, 2016 [54]	Retro.	10	23–50, 31	Siemens, 1.5	HR/LR 3D GRE (PDW)	Axial	7.34/4.77, 2.39	NA	NA	NA	4	Sentinelle variable coil geometry	Semi-automated (In-house software)
					HR/LR3D GRE (T1W)	Axial	7.34/4.77, 2.39	NA	NA	NA	25	Sentinelle variable coil geometry	
					LR 2D SE (T1W)	Axial	500/12	NA	7.0	NA	NA	Sentinelle variable coil geometry	
Wengert, 2015 [55]	Pros.	43	21–71, 38	Siemens, 3.0	Dixon	Axial, 192	NA/6.0, 2.45, 2.67	NA	NA	352 × 352	6	Dedicated 4-channel bilateral	Fully-automated (AUQV)
O’Flynn, 2014 [56]	Retro.	33	(N = 17):	Siemens, 1.5	Fat-suppressed SS-EPI (DWI)	Axial	6300/83	34.0	5.0	NA	NA	Dedicated 4-channel bilateral	Dedicated IDL based software for ADC calculation
			33–49, 43										
			(N = 16):										
			27–49, 40										
Kim, 2016 [57]	Pros.	57	32–74, 50.8	Siemens, 3.0	Fat-suppressed TSE (T2W)	Sagittal	7623/91	22 × 22	3.0	320 × 246	NA	Dedicated 4-breast array	Manually
					Fat-suppressed SS-EPI (DWI)	Axial	5200/74	340 × 179	5.0	80 × 190	NA	Dedicated 4-breast array	
					Fat-suppressed 3D FLASH (T1W)	Sagittal	4.5/1.6	22 × 22	2.0	352 × 292	20	Dedicated 4-breast array	

Abbreviations: Retro.: retrospective; Pros.: prospective; C.S.: cross-sectional; Pre.: pre-menopausal; Post.: post-menopausal; T1W: T1-weighted; T2W: T2-weighted; SPAIR: spectral attenuated inversion recovery; TSE: turbo spin-echo; SGRE: spoiled gradient-echo; VIBRANT: volume image breast assessment; GRE: gradient-echo; FSE: fast spin-echo; STIR: short-TI inversion recovery; DWI: diffusion-weighted imaging; VIBE: volumetric interpolated breath-hold examination; FLASH: fast low angle shot; IDEAL: iterative decomposition of water and fat with echo asymmetry and least squares estimation; HR: high-resolution; LR: low-resolution; PDW: proton density-weighted; SS-EPI: single shot- echo-planar imaging; FCM: fuzzy c-mean clustering algorithm; N3: non-parametric non-uniformity normalization; AUQV: automated user-independent quantitative volumetric.

**Table 2:** Sample size, Mean, and SD of breast volume, fibroglandular volume, and percent of breast density of the (21) included studies in the subgroup meta-analyses.

Author, Year	Breast Volume, BV (cm^3^)		Fibroglandular Volume, FV (cm^3^)			Breast Density, BD (%)	
	Mean	SD	Mean	SD	N	Mean	SD
Chang, 2011 [25]	681	359	100	58	38	17.50	9.50
Nie, 2010 [26]	-	-	104	62	141	15.30	8.10
	-	-	112	73	89	16.70	10.10
Perutz, 2016 [27]	2210	1125	297	128	68	16.60	11.20
Moon, 2018 [28]	537.59	287.74	-	-	89	20.30	8.60
Chen, 2010 [29]	-	-	-	-	35	16.6 0	9.30
Ha, 2016 [30]	-	-	-	-	60	15.30	10.07
Chen, 2016 [31]	537.59	287.74	-	-	23	24.71	15.16
Moon, 2011 [32]	544.90	207.41	-	-	40	23.79	16.62
Klifa, 2010 [11]	-	-	-	-	35	28.0	18.00
Chen, 2011 [1]	358	174	79	66	16	22.10	2.60
Bertrand, 2015 [36]	413.5	364.3	104.2	70.6	182	27.60	20.50
Bertrand, 2016 [37]	418.7	369.3	104.7	70.3	172	27.40	20.00
Dorgan, 2012 [38]	-	-	104.67	71.28	174	28.15	20.39
Chen, 2012 [45]	528	263	117	82	34	24.10	12.40
Choi, 2017 [49]	-	-	-	-	38	14.80	14.40
Chan, 2011 [48]	-	-	-	-	6	8.70	3.40
	-	-	-	-	24	21.20	8.30
Chen, 2013 [50]	-	-	-	-	24	7.50	3.80
Tagliafico, 2014 [3]	-	-	-	-	48	55.00	23.20
Ledger, 2016 [54]	482.6	296.2	135.2	56.2	10	35.40	16.20
Chen, 2013 [53]	-	-	48.1	24.7	24	20.20	7.80
Wengert, 2015 [55]	1462.43	803.38	-	-	43	26.05	19.47

**Table 3:** Study size (N), mean, SD, coefficient of variations (CV), and cluster membership of the included studies.

Study Code	Author, Year	N	Mean	SD	CV	Cluster Membership
P1.01	Chang, 2011 [25]	38	17.50	9.50	54.29	1
P1.04	Nie, 2010 [26]	89	16.70	10.10	60.48	1
P1.05	Pertuz, 2016 [27]	68	16.60	11.20	67.47	1
P1.06	Moon, 2018 [28]	89	20.30	8.60	42.36	1
P1.07	Chen, 2010 [29]	35	16.60	9.30	56.02	1
P1.08	Ha, 2016 [30]	60	15.30	10.07	65.82	1
P4.13	Choi, 2017 [49]	38	14.80	14.40	97.30	1
P4.15	Chan, 2011 [48]	24	21.20	8.30	39.15	1
P6.03	Chen, 2013 [53]	24	20.20	7.80	38.61	1
P1.09	Chen, 2016 [31]	23	24.71	15.16	61.35	2
P1.10	Moon, 2011 [32]	40	23.79	16.62	69.86	2
P1.11	Klifa, 2010 [11]	35	28.00	18.00	64.29	2
P3.01	Bertrand,2015 [36]	182	27.60	20.50	74.28	2
P3.02	Bertrand, 2016 [37]	172	27.40	20.00	72.99	2
P3.03	Dorgan, 2012 [38]	174	28.15	20.39	72.43	2
P4.03	Chen, 2012 [45]	34	24.10	12.40	51.45	2
P6.05	Wengert, 2015 [55]	43	26.05	19.47	74.74	2
P1.12	Chen, 2011 [1]	16	22.10	2.60	11.76	3
P4.14	Chan, 2011 [48]	6	8.70	3.40	39.08	4
P4.16	Chen, 2013 [50]	24	7.50	3.80	50.67	4
P5.04	Tagliafico, 2014 [3]	48	55.00	23.20	42.18	5
P5.08	Ledger, 2016 [54]	10	33.40	16.20	45.76	6
